# Transfer learning–based PET/CT three-dimensional convolutional neural network fusion of image and clinical information for prediction of EGFR mutation in lung adenocarcinoma

**DOI:** 10.1186/s12880-024-01232-5

**Published:** 2024-03-04

**Authors:** Xiaonan Shao, Xinyu Ge, Jianxiong Gao, Rong Niu, Yunmei Shi, Xiaoliang Shao, Zhenxing Jiang, Renyuan Li, Yuetao Wang

**Affiliations:** 1 Department of Nuclear Medicine, The Third Affiliated Hospital of Soochow University, Changzhou, 213003 China; 2 Institute of Clinical Translation of Nuclear Medicine and Molecular Imaging, Soochow University, Changzhou, 213003 China; 3 Department of Radiology, The Third Affiliated Hospital of Soochow University, Changzhou, 213003 China; 4 Interdisciplinary Institute of Neuroscience and Technology, School of Medicine, Zhejiang University, Hangzhou, 310009 China; 5 Sir Run Run Shaw Hospital, School of Medicine, Zhejiang University, Hangzhou, 310058 China

**Keywords:** Lung adenocarcinoma, Positron emission tomography/computed tomography, Deep learning; radiomics, Epidermal growth factor receptor

## Abstract

**Background:**

To introduce a three-dimensional convolutional neural network (3D CNN) leveraging transfer learning for fusing PET/CT images and clinical data to predict EGFR mutation status in lung adenocarcinoma (LADC).

**Methods:**

Retrospective data from 516 LADC patients, encompassing preoperative PET/CT images, clinical information, and EGFR mutation status, were divided into training (*n* = 404) and test sets (*n* = 112). Several deep learning models were developed utilizing transfer learning, involving CT-only and PET-only models. A dual-stream model fusing PET and CT and a three-stream transfer learning model (TS_TL) integrating clinical data were also developed. Image preprocessing includes semi-automatic segmentation, resampling, and image cropping. Considering the impact of class imbalance, the performance of the model was evaluated using ROC curves and AUC values.

**Results:**

TS_TL model demonstrated promising performance in predicting the EGFR mutation status, with an AUC of 0.883 (95%CI = 0.849–0.917) in the training set and 0.730 (95%CI = 0.629–0.830) in the independent test set. Particularly in advanced LADC, the model achieved an AUC of 0.871 (95%CI = 0.823–0.919) in the training set and 0.760 (95%CI = 0.638–0.881) in the test set. The model identified distinct activation areas in solid or subsolid lesions associated with wild and mutant types. Additionally, the patterns captured by the model were significantly altered by effective tyrosine kinase inhibitors treatment, leading to notable changes in predicted mutation probabilities.

**Conclusion:**

PET/CT deep learning model can act as a tool for predicting EGFR mutation in LADC. Additionally, it offers clinicians insights for treatment decisions through evaluations both before and after treatment.

**Supplementary Information:**

The online version contains supplementary material available at 10.1186/s12880-024-01232-5.

## Background

Non-small cell lung cancer (NSCLC) accounts for nearly 85% of primary lung cancers, and adenocarcinoma (ADC) is the most common subtype [1]. In Asia, epidermal growth factor receptor (EGFR) mutations are found in up to 50% of ADC patients [2]. In recent decades, tyrosine kinase inhibitors (TKI) have been shown to prolong progression-free survival and improve the quality of life in patients with EGFR mutations, especially those with advanced ADC [3]. Currently, molecular pathology is the gold standard for determining EGFR mutation status, but there are limitations. The limitations include sampling bias due to tumor heterogeneity, the requirement for invasive biopsies and related complications, slow detection speed, potentially high costs, and the possibility of unreliable results due to insufficient quantity or quality of tissue [4]. Additionally, during the course of disease treatment and progression, the status of EGFR mutations and the immune landscape may change [5]. Therefore, there is an urgent need for a noninvasive, accurate, simple, and reproducible method to predict EGFR mutations.

18 (^18^F)-fluorodeoxyglucose (FDG) PET/CT is a widely accepted noninvasive method for evaluating NSCLC [6–9]. Two recent meta-analyses confirmed the moderate predictive capability of SUVmax for EGFR mutations, with AUC = 0.68–0.69 [10, 11]. Recent studies have focused on ^18^F-FDG PET/CT radiomics [12, 13]. Heterogeneity is more likely present in tumors with EGFR mutations [12, 14], which radiomics may capture. However, PET/CT-based radiomics features showed remarkable predictive power for EGFR mutations (AUC = 0.50–0.87) [15], yet its clinical application requires further investigation for confirmation and optimization.

Convolutional neural networks (CNNs) have performed well in lesion detection, segmentation, and classification [16–18]. Few studies have used PET/CT deep learning models to predict EGFR mutations, mainly due to the data paucity. The only two PET/CT studies with deep learning focused on two-dimensional CNN were trained from scratch [19, 20]; although this method reduces the processing burden, it inevitably affects its performance. Transfer learning (TL) from the pretrained model using ImageNet data has been the standard for deep learning in medical imaging [21]. This method, however, has two limitations: first, the input to the model must be two-dimensional (2D), and thus the rich anatomical three-dimensional (3D) medical images are lost; second, due to the great difference between medical images and natural images, the performance of TL from natural images to medical images is not obvious. To overcome these limitations, we employed Models Genesis, a pretrained model specifically designed for 3D medical imaging data [22]. Unlike other transfer learning strategies, such as pretraining through proxy tasks like lung nodule segmentation or supervised metric learning networks [23], Models Genesis utilizes a self-supervised learning strategy. It focuses on learning from 3D image information to better utilize the spatial information inherent in 3D, demonstrating superiority across multiple 3D medical imaging tasks.

Deep learning offers unique advantages in enhancing the accuracy of medical image diagnosis, especially when integrated with clinical data [24, 25]. These studies underscore the importance of clinical information in constructing efficient deep learning predictive models, highlighting the value of clinical data in understanding and predicting the complex biological influences on EGFR mutation status in lung adenocarcinoma. In this study, we developed a multimodal deep learning model based on ^18^F-FDG PET/CT, which fully harnesses the inherent 3D characteristics of medical imaging to align more closely with actual clinical scenarios, potentially enhancing the accuracy and reliability of EGFR mutation prediction. This integrative approach provides a comprehensive fusion of radiological and clinical data [26], and also enables efficient classification of EGFR mutation status.

## Methods

### Adherence to checklists

This study adhered to the CLEAR checklist [27] for the reporting of our radiomics research. The completed CLEAR checklist has been listed in Table S1. To ensure the transparency and reproducibility of our study, we have made all the raw data and analysis code publicly available. For detailed links, please refer to Availability of data and materials.

### Participants

**Fig. 1:**
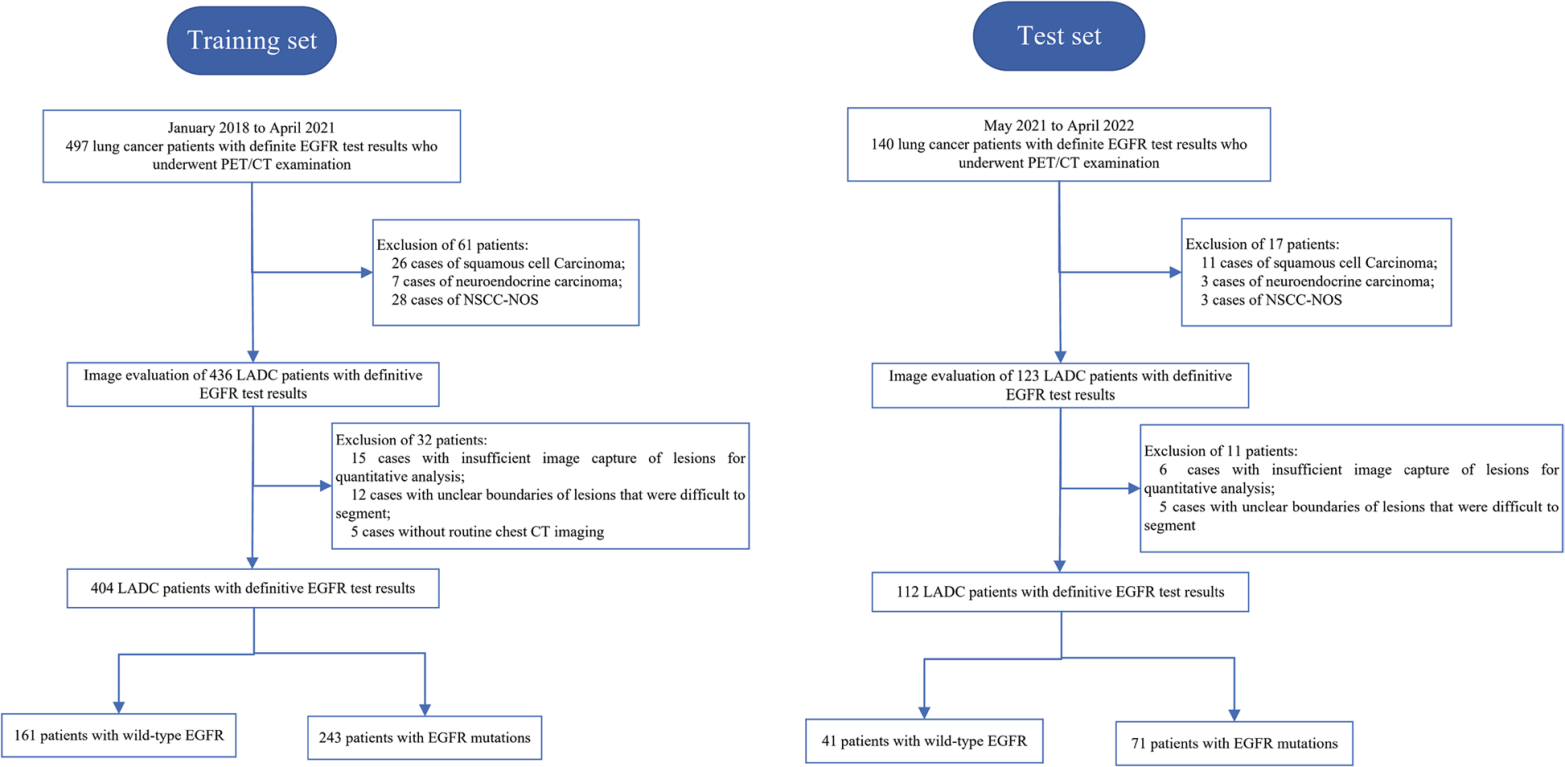
Flowchart of patient enrollment. LADC, lung adenocarcinoma; NSCC-NOS, non‑small cell carcinoma‑not otherwise specified

This retrospective single-center cohort study used privately sourced data, obtaining information from consecutive patients at the Third Affiliated Hospital of Soochow University. Patients with histologically confirmed lung cancer underwent pretreatment ^18^F-FDG PET/CT scans in our department between January 2018 and April 2022. The Institutional Review Board approved this study (No. [2022] KD 087) and waived the need for informed consent from the patients. All patient data used in this study were fully de-identified to ensure privacy and confidentiality, in accordance with international data protection guidelines and standards. The sample size was determined based on the consecutive patients available during the study period. Inclusion criteria: (1) the patient was confirmed with lung cancer by surgery or pathological biopsy; (2) the patient underwent ^18^F-FDG PET/CT examination before surgery, and the interval between surgery and examination was less than 30 days; (3) the patient had definite EGFR test results; and (4) the patient had no history of other malignant tumors. Exclusion criteria: (1) other pathological subtypes except for ADC, (2) lesions with poor image quality or difficulty in measurement, (3) absence of routine chest CT imaging, and (4) severe liver disease or diabetes. Using an internal testing technique, we designated 404 patients from January 2018 to April 2021 as the training set, while the independent test set was formed from 112 patients spanning May 2021 to April 2022. The process of patient enrollment is outlined in Fig. 1. All data used in this study have not been published or used in any other previous publications.

In this study, we also collected clinical characteristics of the patients, which included: age, gender, smoking history, type of nodules, location of nodules, tumor size, clinical stage (I-IV), carcinoembryonic antigen (CEA), and maximum standardized uptake value (SUVmax).

### EGFR mutation detection

The detection of EGFR mutations was conducted in tissue samples obtained by surgical resection or puncture. Mutations in exons 18–21 of the EGFR gene were detected using real-time fluorescent PCR. Real-time fluorescent PCR was conducted per the instructions of the Shanghai Yuanqi EGFR gene mutation detection kit, and the details are described in the Supplementary Material EGFR mutation detection method. EGFR mutation was defined if a mutation was detected in any of those exons; otherwise, wild-type EGFR was defined.

### FDG PET/CT image acquisition

The image acquisition protocol was described according to an acquisition protocol based on the Imaging Biomarker Standardization Initiative (IBSI) reporting guidelines [28]. The image acquisition parameters are listed in Table S2. The patient underwent non-contrast chest CT imaging and ^18^F-FDG PET/CT (Biograph mCT 64, Siemens, Erlangen, Germany) within one month before surgical treatment. Before the administration of ^18^F-FDG, the blood glucose levels of the patients were checked to ensure they were within the acceptable range (< 150 mg/dL). According to the European Association of Nuclear Medicine (EANM) guidelines 1.0 (version 2.0, published in February 2015) [29], ^18^F-FDG PET/CT images were acquired 60 ± 5 min after ^18^F-FDG injection. All PET/CT images were reconstructed on a processing workstation (TrueD software, Siemens Healthcare).

### Image segmentation and pre-processing

A nuclear medicine physician with over 10 years of experience selected regions of interest on PET and CT images, and all images were segmented using 3D-Slicer (version 4.11.20200930, www.slicer.org). For CT images (3 mm), we utilized a semi-automatic method with NVIDIA AI-assisted annotation (3D-Slicer built-in) and a boundary-based CT segmentation model to process the lung nodule images. For PET images, 3D masks were generated using a semi-automatic segmentation method developed by Beichel et al. [30]. Please refer to Supplementary Material-PET/CT image pre-processing for deep learning for details.

### Development of the deep learning models

**Fig. 2:**
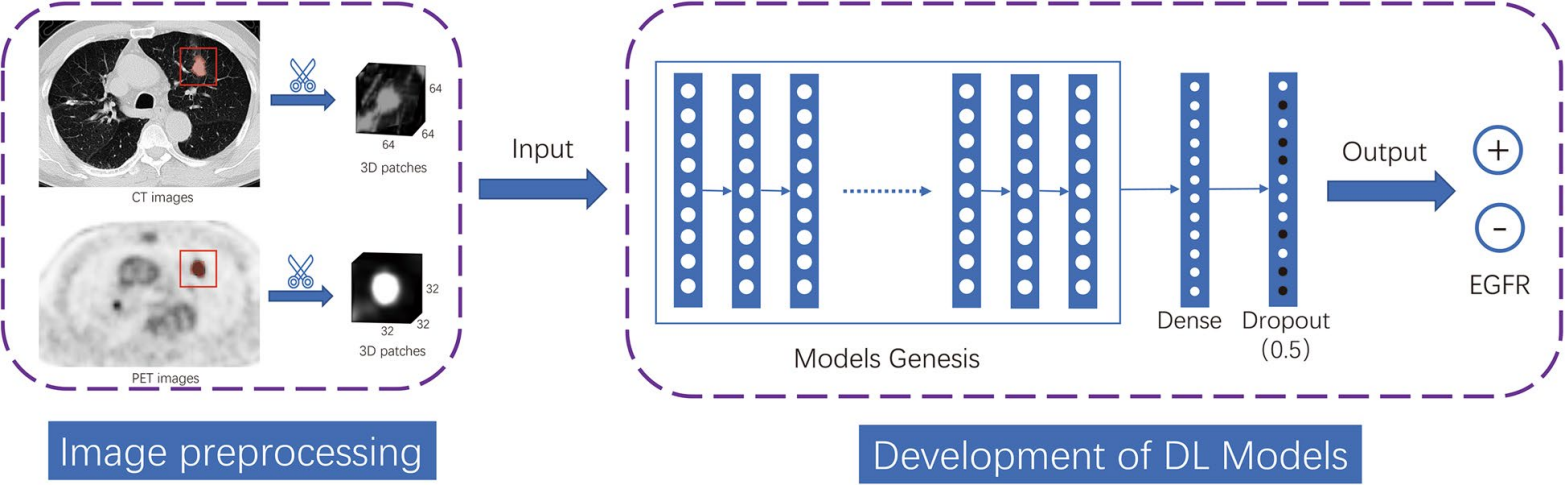
Overall pipeline for deep learning model development

The overall approach to developing the deep learning model is summarized in Fig. 2. To initialize the encoder for the target classification task, we employed Models Genesis, an openly available deep learning model on GitHub (https://github.com/MrGiovanni/ModelsGenesis.git). We subsequently fine-tuned it in accordance with the specific requirements of our target task. The structure diagrams of four 3D CNNs, namely CT TL (CT_TL), PET TL (PET_TL), dual-stream TL (DS_TL, fusing PET and CT), and three-stream TL (TS_TL, adding clinical data to PET and CT) networks, are depicted in Fig. S1. Additionally, we trained two models from scratch (CT_origin and PET_origin). Details of the model training and tuning are described in the Supplementary Material-Training of deep learning models.

### Visualization of deep learning models

A visualization method, Grad-CAM, was adopted to explain the prediction process of the deep learning models [31]. High-reaction regions (predicted tumor-associated areas) were retained with a cutoff value of 0.5. When the deep learning model predicts the EGFR mutation status, it informs the clinician which areas attracted the model's attention (divided into wild and mutant type-associated activation areas). We selected only 3D input cross-sectional intermediate layer images for visualization.

### Statistical analysis

Statistical analysis of clinical data and routine PET/CT metabolic parameters was performed using R software (version 3.4.3; http://www.R-project.org/). The model's performance was assessed using receiver operating characteristic (ROC) curves and quantified by calculating area under the receiver operating characteristic curve (AUC) and 95% confidence intervals (CI). In addition, we further evaluated the model by calculating accuracy, sensitivity, specificity, positive predictive value (PPV), and negative predictive value (NPV) to obtain comprehensive quantitative performance metrics. Due to the presence of class imbalance in the data, we chose AUC as the primary performance metric for the model. A pairwise comparison of the AUC values of the models was performed using the method proposed by Delong et al. [32]. All statistical tests were two-sided, and statistical significance was interpreted as *p* < 0.05. In this study, no missing data was encountered.

## Results

### Clinical characteristics of patients

Table 1 describes the clinical characteristics of 516 patients, of which 202 (39.1%) were with wild-type EGFR and 314 (60.9%) exhibited EGFR mutations. During our analysis, we observed significant differences in clinical characteristics such as gender, smoking history, type of nodules, tumor long axis, and tumor short axis between patients with EGFR mutations and those with wild-type EGFR (with *p* < 0.05 in both the training and test sets). Based on these observations and informed by clinical prior knowledge [33, 34], we decided to incorporate these characteristics as clinical information in constructing TS_TL based on PET/CT images. During this process, we normalized the quantitative indicators to ensure they fall within the range of 0 to 1, aligning with the scale of the normalized PET/CT image data. For categorical variables, we retained their original binary format, allowing for the consideration of clinical variable diversity in the construction of the TS_TL model.

**Table 1:** Clinical characteristics of patients with different EGFR mutation statuses in the training and test sets

	Training Set *n* = 404			Test Set *n* = 112		
EGFR	Wild-type	Mutation	*p*-value	Wild-type	Mutation	*p*-value
N	161	243		41	71	
Age (years)	64.8 (9.1)	63.7 (9.2)	0.210	64.2 (8.8)	63.2 (9.4)	0.556
Gender			< 0.001			0.018
Female	51 (31.7%)	162 (66.7%)		13 (31.7%)	39 (54.9%)	
Male	110 (68.3%)	81 (33.3%)		28 (68.3%)	32 (45.1%)	
Smoking history	87 (54.0%)	54 (22.2%)	< 0.001	18 (43.9%)	16 (22.5%)	0.018
Type of nodules			< 0.001			0.030
Solid	128 (79.5%)	147 (60.5%)		32 (78.1%)	41 (57.8%)	
Subsolid	33 (20.5%)	96 (39.5%)		9 (22.0%)	30 (42.3%)	
Location of nodules			0.645			0.662
Upper right	47 (29.2%)	81 (33.3%)		10 (24.4%)	21 (29.6%)	
Middle right	6 (3.7%)	14 (5.8%)		4 (9.8%)	8 (11.3%)	
Lower right	34 (21.1%)	50 (20.6%)		12 (29.3%)	14 (19.7%)	
Upper left	44 (27.3%)	63 (25.9%)		9 (22.0%)	21 (29.6%)	
Lower left	30 (18.6%)	35 (14.4%)		6 (14.6%)	7 (9.9%)	
Tumor long axis (mm)	32.1 (20.7–47.2)	25.6 (20.3–37.6)	0.002	40.7 (29.5–49.5)	29.4 (23.5–38.3)	0.006
Tumor short axis (mm)	23.2 (14.6–33.2)	19.0 (14.3–27.7)	0.015	29.6 (24.1–33.4)	22.2 (16.0–28.4)	0.006
Clinical stage			0.005			0.228
I	52 (32.3%)	125 (51.4%)		8 (19.5%)	25 (35.2%)	
II	12 (7.5%)	3 (1.2%)		4 (9.8%)	5 (7.0%)	
III	33 (20.5%)	35 (14.4%)		8 (19.5%)	9 (12.7%)	
IV	64 (39.8%)	80 (32.9%)		21 (51.2%)	32 (45.1%)	
CEA (ng/ml)	5.24 (2.61–15.61)	3.23 (1.60–12.25)	0.016	5.08 (2.42–13.39)	5.28 (2.09–18.68)	0.880
SUVmax	13.03 (6.27–18.21)	10.14 (3.44–17.51)	0.005	15.60 (8.51–20.67)	13.55 (4.85–17.97)	0.114

Data in the table were expressed as Mean (SD) or Median (Q1-Q3) / N (%)

### Diagnostic validation of several deep learning models

**Fig. 3:**
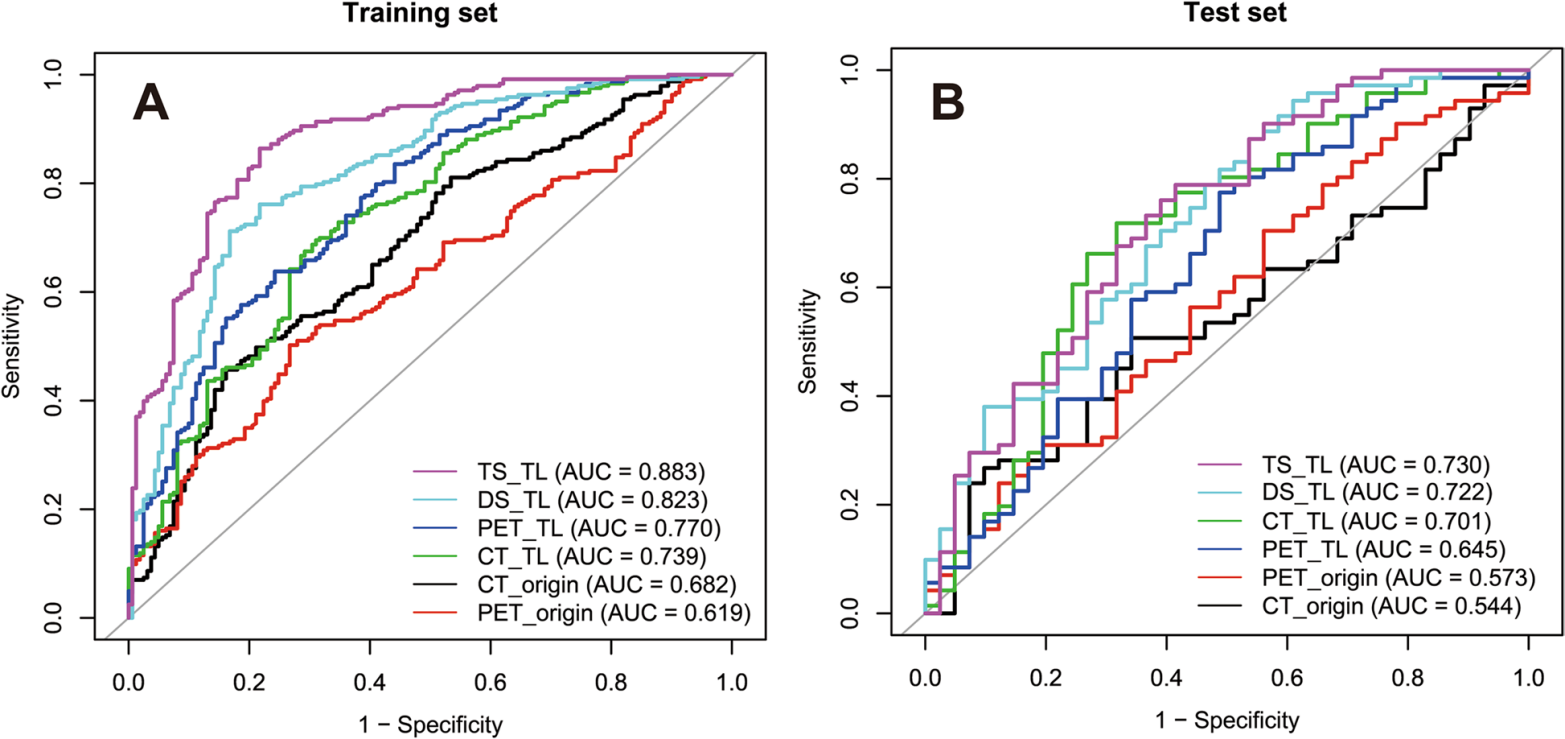
ROC curves of different transfer learning models in the training and test sets. AUC, area under the receiver operating characteristic curve; CT_origin, CT model from scratch; CT_TL, CT transfer learning; PET_origin, PET model from scratch; PET_TL, PET transfer learning; DS_TL, dual-stream transfer learning; TS_TL, three-stream transfer learning

The predictive performance of several deep learning models in the test set is listed in Table 2 (see Table S3 for training set). In the training set, TS_TL showed the best predictive performance (AUC = 0.883), further confirmed in the independent test set (AUC = 0.730). CT_TL and PET_TL outperformed CT_origin and PET_origin in both training and test sets, with significant improvement in test set for CT_TL (AUC = 0.701 vs. 0.544, p = 0.027) and in training set for PET_TL (AUC = 0.770 *vs*. 0.619, *p* < 0.001; Fig. 3 A, B). Also, CT_TL showed high sensitivity and low specificity in both sets, while PET_TL showed low sensitivity and high specificity (Table S3 and Table 2).

**Table 2:** Predictive performance of several deep learning models in the test set

Model	AUC (95%CI)	Accuracy	Sensitivity	Specificity	PPV	NPV
CT_origin	0.544 (0.435–0.653)	0.536	0.507	0.585	0.679	0.407
CT_TL	0.701 (0.595–0.808)	**0.688**	**0.746**	0.585	0.757	**0.571**
PET_origin	0.573 (0.461–0.684)	0.536	0.521	0.561	0.673	0.404
PET_TL	0.645 (0.534–0.756)	0.589	0.549	**0.659**	0.736	0.458
DS_TL	0.722 (0.622–0.822)	0.661	0.676	0.634	0.762	0.531
TS_TL	**0.730 (0.629–0.830)**	0.670	0.676	**0.659**	**0.774**	0.540

Bold numbers indicate the best results for each evaluation metric

*AUC* Area under the receiver operating characteristic curve, *PPV* positive predictive value, *NPV* Negative predictive value, *CT_origin* CT model from scratch, *CT_TL* CT transfer learning, *PET_origin* PET model from scratch, *PET_TL* PET transfer learning, *DS_TL* dual-stream transfer learning, *TS_TL* three-stream transfer learning

The tumor long axis, tumor short axis, tumor stage, and SUVmax showed statistical differences between the two sets (all *p* < 0.05), which might be attributed to the different compositions of patients at different periods in our center (Table S4). To eliminate this discrepancy, we performed a hierarchical analysis to validate the diagnostic performance of the four TL models for different tumor stages (Table S5). For stage I-II tumors, TS_TL exhibited significant overfitting (AUC of training set *vs*. test set: 0.903 *vs*. 0.667), while DS_TL had the optimal performance in the test set (AUC of training set *vs*. test set: 0.862 *vs*. 0.708); for stage III-IV tumors, TS_TL showed the best performance in both training and test sets (AUC of training set *vs*. test set: 0.871 *vs*. 0.760).

### Comparisons of deep learning models and radiomics models

We established four radiomics models of CT, PET, PET/CT, and PET/CT combined with clinical features (CT_RS, PET_RS, DS_RS, TS_RS) and compared them with the proposed TL models (Table 3 and Table S6). The specific methods for the development of the radiomics model are described in the Supplementary Material-Development of four radiomics models, Table S7 and Table S8. ROC curves of the four radiomics models and SUVmax are shown in Figure S2. First, PET_RS outperformed SUVmax in both training and test sets but only showed significance in the training set (training set: AUC = 0.651 *vs*. 0.582, *p* = 0.014). It was subsequently validated that PET_TL performed significantly better in the training set but slightly inferior in the test set than the PET_RS model (training set: AUC = 0.770 *vs*. 0.651, *p* < 0.001; test set: AUC = 0.645 *vs*. 0.661, *p* = 0.763). CT_TL performed better than CT_RS in both training and test sets, with a significant improvement in the training set only (training set: AUC = 0.739 *vs*. 0.655, *p* < 0.001).

**Table 3:** Comparison of the prediction performance of deep learning models and radiomics models in the test set

Model	AUC (95%CI)	Accuracy	Sensitivity	Specificity	PPV	NPV
CT_RS	0.639 (0.529–0.749)	0.652	**0.887**	0.244	0.670	0.556
CT_TL	0.701 (0.595–0.808)	**0.688**	0.746	0.585	0.757	**0.571**
PET_RS	0.661 (0.552–0.769)	0.643	0.676	0.585	0.738	0.511
PET_TL	0.645 (0.534–0.756)	0.589	0.549	0.659	0.736	0.458
DS_RS	0.620 (0.509–0.730)	0.670	0.831	0.390	0.702	**0.571**
DS_TL	0.722 (0.622–0.822)	0.661	0.676	0.634	0.762	0.531
TS_RS	0.711 (0.613–0.809)	0.616	0.577	**0.683**	0.759	0.483
TS_TL	**0.730 (0.629–0.830)**	0.670	0.676	0.659	**0.774**	0.540

Bold numbers indicate the best results for each evaluation metric

*AUC* Area under the receiver operating characteristic curve, *PPV* Positive predictive value, *NPV* Negative predictive value, *CT_RS* CT radiomics, *CT_TL* CT transfer learning, *PET_RS* PET radiomics, *PET_TL* PET transfer learning, *DS_RS* PET/CT radiomics, *DS_TL* dual-stream transfer learning, TS_RS PET/CT radiomics combined with clinical features, *TS_TL* three-stream transfer learning

Compared to single-modal radiomics models CT_RS and PET_RS, DS_RS performed slightly better in the training set and worse in the test set, but the differences were not significant (training set: AUC = 0.662 *vs*. 0.655 *vs*. 0.651, both *p* > 0.05; test set: AUC = 0.620 *vs*. 0.639 *vs*. 0.661, both *p* > 0.05). DS_TL performed better than DS_RS in both training and test sets (training set: AUC = 0.823 *vs*. 0.662, *p* < 0.001; test set: AUC = 0.722 *vs*. 0.620, *p* = 0.033).

By combining DS_RS with clinical features, TS_RS outperformed CT_RS, PET_RS, and DS_RS in both training and test sets, while statistical significance was only noted in the training set (training set: AUC = 0.771 *vs*. 0.655 *vs*. 0.651 *vs*. 0.662, all *p* < 0.001). Similarly, TS_TL outperformed TS_RS in both training and test sets, with statistical significance in training set only (training set: AUC = 0.883 *vs*. 0.771, *p* < 0.001). When DS_TL was combined with clinical features, TS_TL outperformed DS_TL in both training and test sets, with significance shown only in the training set (training set: AUC = 0.883 *vs*. 0.823, *p* < 0.001).

### TS_TL predicted tumor-associated areas for solid or subsolid lesions of different mutation subtypes

**Fig. 4:**
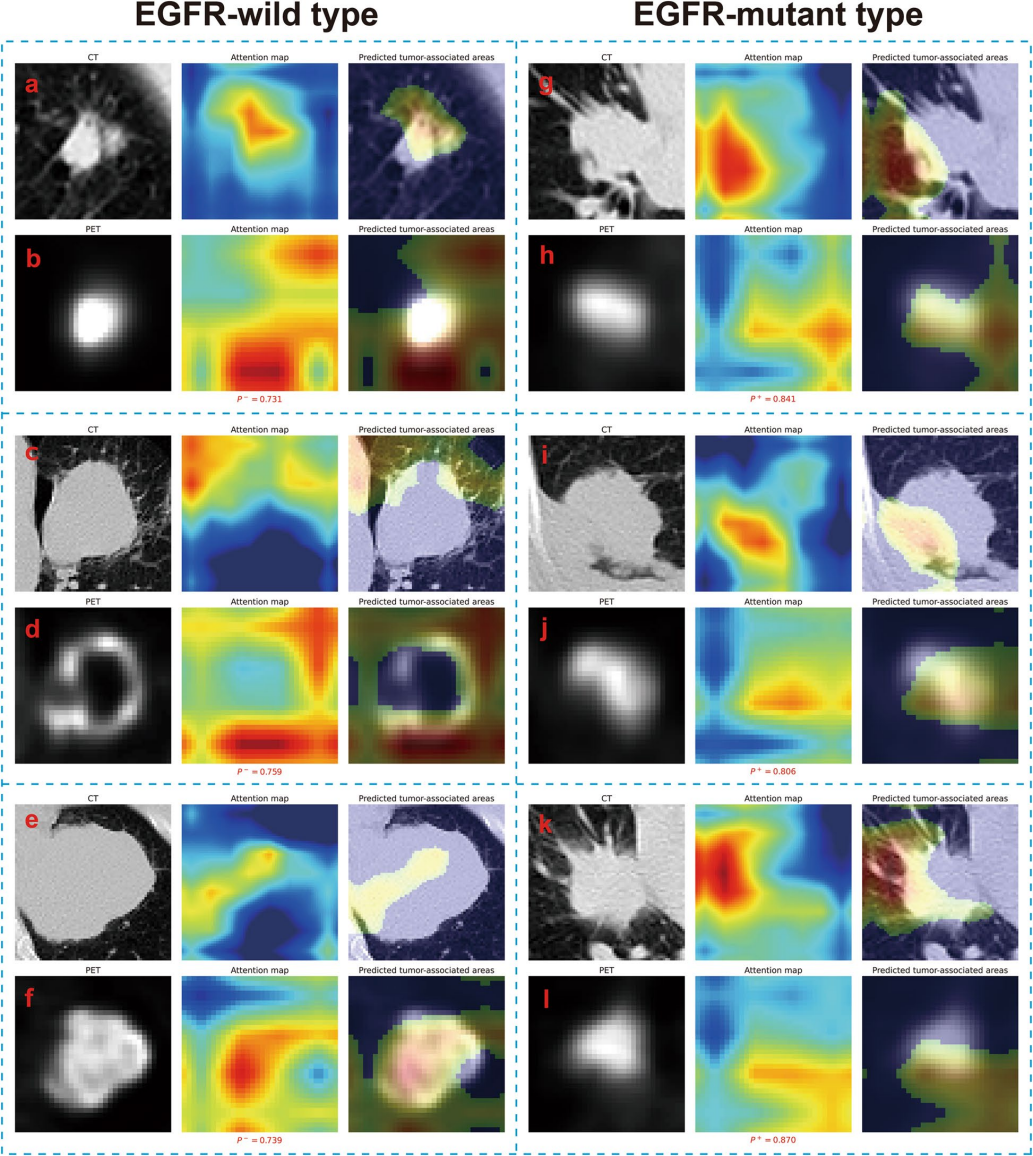
TS_TL predicted tumor-associated areas for solid lesions with either EGFR wild-type or mutation. For each submap, the input CT or PET image, the attention map, and the model-predicted tumor-associated areas are from left to right. For LADC tumors, the deep learning model generated an attention map indicating the importance of each part of the tumor; high-reaction regions (predicted tumor-associated areas) were retained with a cutoff value of 0.5. *P*^*−*^ and *P*^+^ represented the predicted probability of EGFR wild-type and mutant, respectively

Figure 4 displays six representative solid lesions (three wild-type and three mutant) along with TS_TL's wild and mutant type-associated activation areas. In solid lesions, TS_TL consistently focuses on the local and peripheral areas of the lesion in CT images (Fig. 4a, c, e, g, i, k) and most metabolic areas in PET images (Fig. 4b, d, f, h, j, l), regardless of wild-type or mutant status.

**Fig. 5:**
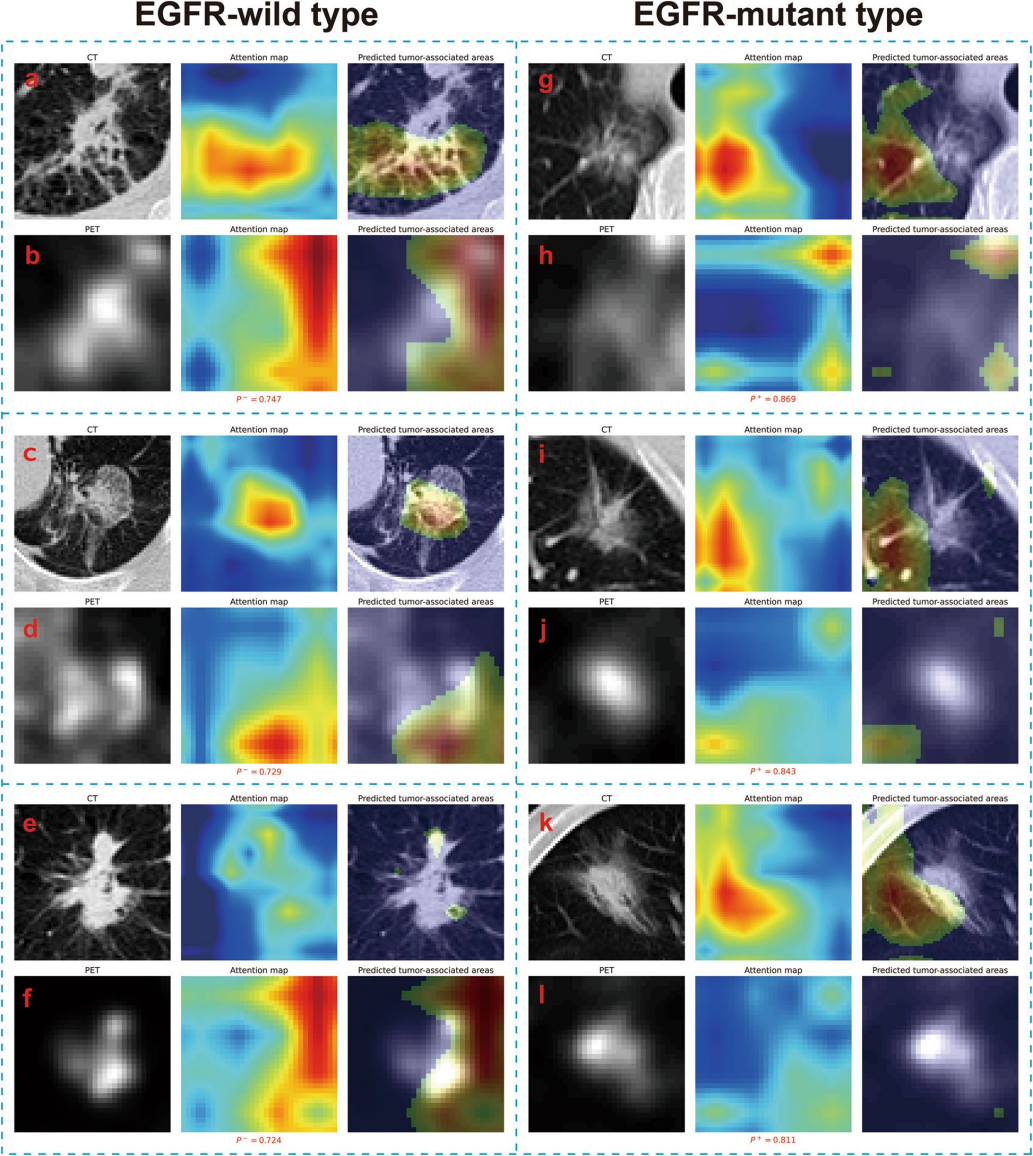
TS_TL predicted tumor-associated areas for subsolid lesions with either EGFR wild-type or mutation. For each submap, the input CT or PET image, the attention map, and the model-predicted tumor-associated areas are from left to right. For LADC tumors, the deep learning model generated an attention map indicating the importance of each part of the tumor; high-reaction regions (predicted tumor-associated areas) were retained with a cutoff value of 0.5. *P*^*−*^ and *P*^+^ represented the predicted probability of EGFR wild-type and mutant, respectively

Figure 5 displays six representative subsolid lesions (three wild-type and three mutant) along with TS_TL's wild and mutant type-associated activation areas. For subsolid wild-type lesions, the wild type-associated activation areas in CT and PET images are similar to those in solid lesions (Fig. 5a-f). For subsolid mutant lesions, the mutant type-associated activation areas in CT images still robustly capture the local and peripheral areas of the lesion (Fig. 5g, i, k), while the mutant type-associated activation areas in PET images do not effectively capture the metabolic areas of the lesion (Fig. 5, j, l).

### Changes in mutant type-associated activation areas before and after TKI treatment

**Fig. 6:**
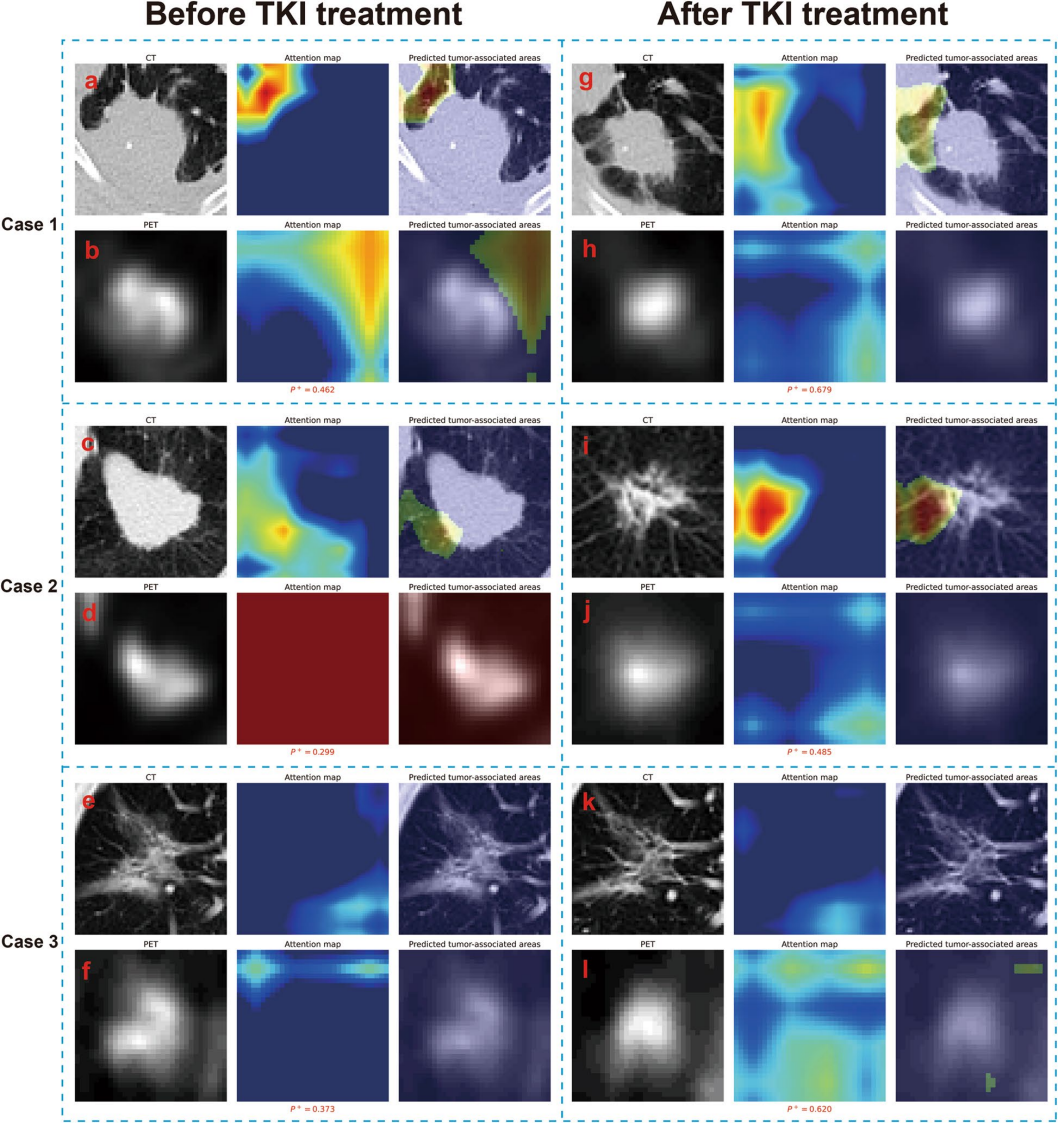
The mutant type-associated activation areas of the TS_TL in three EGFR-mutant lesions before and after TKI treatment. Case 1 (stage IV): A female non-smoker in the 60–65 age range presented with a solid mass in the left lower lung (41.1 × 29.7 mm) with an EGFR mutation in exon 20 and received oral poziotinib treatment; through PET/CT re-examination after 21 months, the original lesion shrunk (30.2 × 24.6 mm) and its metabolism was lower than before (SUVmax from 14.8 to 10.2). Poziotinib is a novel targeted drug for rare insertion mutations in exon 20 of EGFR and HER2. Case 2 (stage IV): A male smoker in the 65–70 age range presented with a solid mass in the left upper lung (40.9 × 26.7 mm) harboring an exon 21 EGFR mutation. The patient received oral Osimertinib treatment, and after 6 months, a follow-up PET/CT examination revealed a significant reduction in the size of the original lesion, which became subsolid (20.7 × 15.9 mm), and a decrease in metabolism (SUVmax from 11.8 to 3.9). Case 3 (stage IV): A male non-smoker in the 80–85 age range presented with a subsolid mass in the right upper lung (35.5 × 30.5 mm) carrying an exon 19 EGFR mutation. The patient received oral Icotinib hydrochloride treatment, and after 46 months, a follow-up PET/CT examination revealed a slight reduction in the size of the original lesion (28.5 × 27.9 mm) and a decrease in metabolism (SUVmax from 3.1 to 1.9)

Figure 6 shows the changes in the mutant type-associated activation areas and the predicted mutation probability (*P*^+^) before and after TKI treatment in three EGFR-mutant patients with effective TKI treatment. Both case 1 and case 2 are solid lesions with non-ideal model predictions before TKI treatment (*P*^+^ values in Fig. 6a, b and c, d are both less than 0.5, indicating that the model has not yet captured a clear image mutation pattern). The *P*^+^ values of both cases significantly increase after TKI treatment (from 0.462 to 0.679 and from 0.299 to 0.485). Looking at the mutant type-associated activation areas after treatment (Fig. 6g, h and i, j), they are also similar to the previously mentioned subsolid mutant lesions (refer to Fig. 5g-l), indicating that the treatment has changed the image pattern captured by the model. For subsolid lesion case 3, there are no obvious mutant type-associated activation areas in either CT or PET pathways before and after treatment (Fig. 6e, f and k, l). However, the *P*^+^ value increases significantly after treatment (from 0.373 to 0.620). The changes in classical methods and *P*^+^ values before and after TKI treatment in 3 cases can be seen in Table S9.

## Discussion

The development of multimodal biomarkers will be a trend in the field of precision oncology in the future [26]. Anatomical information represented by CT and functional information represented by PET are naturally complementary, and the integration of FDG PET/CT images maintains the sensitivity of CT and the specificity of PET [35]. In our study, CT_TL and PET_TL showed high sensitivity and high specificity, resulting in improved performance of DS_TL, particularly in predicting EGFR in early LADC. This might be attributed to the fact that the important regions available for accurately predicting EGFR mutations could be better and more easily localized using metabolic and anatomical information reflected by PET and CT images, respectively [19].

Many studies have revealed clinical factors associated with EGFR mutation in LADC, such as gender, smoking history, and the presence of ground glass opacity (GGO) [33]. Before CNN training, we standardized the CT and PET images through preprocessing, including resampling to match the CNN's input size. This process may partially lose the original lesion size information, so to compensate, we specifically included tumor long and short axis measurements as additional clinical features into the DS_TL model. This is aimed at preserving crucial information about tumor size, ensuring the model fully considers the actual dimensions of the lesion. By adding gender, smoking history, nodule type, and metadata [34], the performance of the TS_TL model has been improved, especially in predicting advanced LADC.

Previous research has shown that radiomic features extracted from PET/CT and CT images can predict gene expression patterns and EGFR mutation status [14, 36]. In their review, Ge et al. [37] highlighted that machine learning-based radiomics (MLR, specifically shallow learning), have demonstrated high accuracy in predicting EGFR mutations. The advantage of CT_TL and PET_TL over the radiomics models in this study was insignificant, possibly due to the small sample size and different patient compositions in the training set. We further developed the DS_RS model through feature fusion, the performance of which in the test set was lower than expected, while DS_TL better integrated the information of two modes, CT and PET, yielding better generalization. Furthermore, we also found that the input of clinical information significantly improved the diagnostic performance of DS_RS, while the performance of DS_TL showed a mild improvement, which could be explained by performance saturation.

For predicting mutation in solid lesions, both CT and PET images play a stable role in the model, and similar findings were obtained by Yin et al. [20]. Existing studies have reported that the features of the edge of the lesions, such as spiculation sign [38] and lobulation sign [39], are related to EGFR mutation. TS_TL's attention for solid lesions is focused on the tumor periphery with the aforementioned characteristics. For wild-type predictions in subsolid lesions, both CT and PET images still play a role in TS_TL; however, in mutant predictions, CT images take the lead in the model, while PET images fail to capture the metabolic areas of the lesion effectively. This is related to the generally low metabolism of most mutant-type subsolid lesions [40], which cannot effectively activate specific convolutional kernels.

Studies have shown that PET/CT can be used to monitor TKI treatment reactions and evaluate the prognosis of NSCLC patients [41]. This study found that TKI treatment may change the image patterns captured by the model, and the change in *P*^+^ value before and after treatment may be superior to traditional indicators, providing an indication of therapeutic efficacy. This is because the *P*^+^ value integrates the lesion image and long-short diameter information (which can be considered a weighted combination of these useful factors), closely related to efficacy assessment.

However, this study also comes with its limitations. (1) Being a single-center study, the wider applicability of this model requires external validation, despite our methodical assignment of patients to the training set and independent test set based on time. (2) Some scholars posit that the model's predicted tumor-associated areas could guide clinicians to optimal biopsy locations within the tumor, mitigating the sampling bias due to tumor heterogeneity [18]. Nonetheless, this proposition warrants further exploration through prospective studies, particularly concerning the biological interpretation of the said tumor-associated areas.

## Conclusions

Our end-to-end deep learning model integrates CT, PET, and clinical data to effectively predict EGFR mutation in LADC. This model could also find predicted tumor-associated areas strongly linked to EGFR mutation status and help clinicians make patient treatment decisions through pre- and posttreatment qualitative and quantitative assessment.

### Supplementary Information

**Supplementary Material 1. **

## Data Availability

No datasets were generated or analysed during the current study.

## Abbreviations

EGFR: Epidermal growth factor receptor
LADC: Lung adenocarcinoma
TKI: Tyrosine kinase inhibitors
CNN: Convolutional neural networks
NSCLC: Non-small cell lung cancer
ADC: Adenocarcinoma
FDG: Fluorodeoxyglucose
TL: Transfer learning
2D: Two-dimensional
3D: Three-dimensional
CEA: Carcinoembryonic antigen
SUV: Standard uptake value
DS_TL: Dual-stream transfer learning
TS_TL: Three-stream transfer learning
HU: Hounsfield units
ROC: Receiver operating characteristic
AUC: Area under the receiver operating characteristic curve
PPV: Positive predictive value
NPV: Negative predictive value
