# Supplementary material for: Transfer learning–based PET/CT three-dimensional convolutional neural network fusion of image and clinical information for prediction of EGFR mutation in lung adenocarcinoma
Source: BMC Med Imaging. 2024 Mar 4;24:54. doi: 10.1186/s12880-024-01232-5 (PMC10913633; doi:10.1186/s12880-024-01232-5)
Supplement: Supplementary file 1 — Supplementary Material 1. [file 12880_2024_1232_MOESM1_ESM.docx]

**Supplementary material**

**EGFR mutation detection method**

The DNA was extracted from samples such as paraffin-embedded pathological tissues or sections of patients and amplified on ABI 7300 fluorescence PCR detector. The amplification conditions were: 42℃, 5min; 94℃, 3min; (94℃, 15sec; 60℃, 60sec) for 40 cycles; the reaction volume was 25µL; the fluorescence signal was collected at 60℃ in the second step of the PCR cycle; the detection channel was FAM-TAMRA, and the reference fluorescence was set to none. The computer automatically processed and analyzed the data.

**PET/CT image pre-processing for deep learning**

The PET images were attenuation corrected using CT data, and the corrected PET images were fused with CT images. The respiratory gating technique was not used during the acquisition. The anisotropic resolutions of the CT and PET images were 0.78 × 0.78 × 3.00 mm^3^ and 4.07 × 4.07 × 3.00 mm^3^, respectively. All PET images were converted to standard uptake value (SUV) units by normalizing radioactive concentrations to ^18^F-FDG injection dose and decay-corrected patient weight, and all CT images were converted to lung windows (window width, 1200HU; window level, −600 HU).

For CT images, use a semi-automatic method to generate a 3D mask. We applied linear resampling to the CT images and used the nearest neighbor technique for the masks (both 1 × 1 × 1 mm^3^), generating a 10-mm circumscribed cube covering the entire tumor and its surrounding region, modified the pixel size to 64 × 64 × 64 pixels through cubic interpolation, and finally inputted it into the deep learning models. For PET images, 3D masks were generated using a semi-automatic segmentation method. Also, we applied linear resampling to the PET images and used the nearest neighbor technique for the masks (both 3 × 3 × 3 mm^3^), generating a 12 mm circumscribed cube covering the entire tumor and its surrounding region, resized it to 32 × 32 × 32 pixels by cubic interpolation, and ultimately inputted it into the deep learning models.

**Training of deep learning models**

The preprocessed CT or PET images were inputs into the deep learning model. FDG-PET was converted to SUVbw (units of body weight), and SUV values were linearly mapped from the range 0–20 back to the range 0–1. Likewise, CT images were expressed in Hounsfield units (HU), and HU values were linearly mapped from the range −1000–400 back to the range 0–1. For TL, Models Genesis pretrained weights were used to initialize the classification model. This framework was implemented in Python 3.7.13, Keras 2.3.1, and TensorFlow 1.14.0 on Windows 10, with a supporting NVIDIA GeForce GTX 1070 graphics processing unit.

To reduce overfitting, we used random flips, random rotations by 90°, and random rotations (-15°-15°) and set the probability value *p* = 0.5. The model's training focused on optimizing deep learning model parameters to establish the relationship between PET/CT images and EGFR mutation status (mutant-type: 1 or wild-type: 0). For the four TL models, fine-tuned from scratch was used ([1](#_ENREF_1)). Use sparse_categorical_crossentropy as loss function, use Adam optimizer, initial learning rate = 1e-5, beta_1 = 0.9, beta_2 = 0.999, epsilon = 1e-08, decay = 0.0. For CT_origin and PET_origin, initial learning rate = 1e-3.

Split the training set using stratified 5-fold cross-validation and start training. The epochs were 300 and 600 for training the four TL models and the two models from scratch, respectively. For the training of CT_origin and CT_TL, batch_size = 8, for the training of PET_origin and PET_TL, batch_size = 24, for the training of DS_TL and TS_TL, batch_size = 6. During training, the weights of positive and negative class samples are balanced using the class_weight parameter. The optimal number of epochs for model training was determined by 5-fold cross-validation, CT_origin was 244, PET_origin was 107, CT_TL was 34, PET_TL was 54, DS_TL was 48, and TS_TL was 70. Then, according to the above optimal epochs, trained the final model on the entire training set, observed its performance on the test set, outputted the classification report, and drew the ROC curve. The best model was selected based on the highest AUC performance on the test set.

**Development of four radiomics models (CT_RS, PET_RS, DS_RS, TS_RS)**

**Image segmentation for radiomics** A nuclear medicine physician (Doctor A) with over 10 years of experience selected regions of interest on PET and CT images. Four weeks after completing the ROI for all cases, Doctor A segmented the tumor region again for 300 patients in the training set, among which 70 patients were randomly selected for Doctor B (A nuclear medicine physician with 5 years of experience) to segment. We use the above methods to calculate the intraclass and interclass correlation coefficients.

**Image Pre-processing** **for radiomics** Before feature extraction, the images were normalized and interpolated (sitkBSpline algorithm, B-spline of order 3 interpolation) using Pyradiomics (http://www.radiomics.io/pyradiomics.html, version 3.0.1) so that the isotropic voxel spacing was ration invariant. Feature extraction was performed to compare the image data from different samples. CT images were resampled to 1 × 1 × 1mm^3,^ and PET images were resampled to 3 × 3 × 3mm^3^. The images were discretized by the fixed binwidth method. The binwidth of CT and PET images were 25 and 0.313, respectively. The bin discretization, Laplacian of Gaussian (LOG), and wavelet transform were preprocessed to generate different feature sets. Different sigma values were used for the LOG filter to extract fine, medium, and coarse features, ranging from 0.5 to 5, with a step size of 0.5 ([2](#_ENREF_2)). The wavelet transforms produced eight decompositions per level (applying all possible combinations of high- or low-pass filters in each of the three dimensions, including HHH, HHL, HLH, HLL, LHH, LHL, LLH, and LLL). Pre-processing steps (including discretization, LOG, and wavelet transform) were performed on all shape features, first-order statistics, and textural features.

**Feature Extraction** In the next step, multiple features from different feature classes were extracted. These categories included shape and morphological features (14 shape features), first-order statistics (18 FOS features), gray-level co-occurrence matrix (24 GLCM features), gray-level dependence matrix (14 GLDM features), gray-level run length matrix (16 GLRLM features), gray-level scale zone matrix (16 GLSZM features), and neighboring gray tone difference matrix (5 NGTDM features). Radiomics features (1781 each) of 3D-mask-based CT and PET images were extracted using Pyradiomics (http://www.radiomics.io/pyradiomics.html) version 3.0.1. Additionally, it's worth noting that the Pyradiomics software adheres to the benchmarks/certifications set by IBSI. All other parameters were maintained at their default configurations.

**Feature Selection** First, robust radiomics features were selected using the intraclass and interclass correlation coefficients (ICC > 0.75, based on the single random raters method) only in the training set. Due to a large number of radiomic features and the relatively small case number in this study, the variance method was used to remove features with small variance (threshold = 0.24) to avoid overfitting the model. Secondly, in the training set, the Mann-Whitney U test was used to screen out radiomic features with a *p* < 0.1 that may be associated with EGFR mutation status. After the U test, all selected radiomic features were standardized using the StandardScaler in the training set, which rescales the data to have a mean of zero and a standard deviation of one. This same scaling method was then applied to the test set independently to ensure consistency across the data. Then, the Least Absolute Shrinkage and Selection Operator (LASSO) algorithm was used on the standardized training set data to select the best predictive features. The LASSO algorithm adds an L1 regularization term to the least squares algorithm to avoid overfitting and employs 5-fold cross-validation. The final CT, PET, and PET/CT fusion screened out 8, 4, and 4 (2CT + 2PET) radiomic features, respectively, plus five clinical features (gender, smoking history, type of nodules, tumor long axis, tumor short axis) to construct TS_RS. The continuous clinical features (tumor long and short axis) were standardized to ensure a consistent scale across all features. The specific features of the four radiomics models are shown in **Table S7**.

**Classifier and Modeling** All our analyses, including feature selection and classification, were performed using an internally developed Python framework in the open-source Python library Scikit-Learn ([3](#_ENREF_3)). The filtered predictive features were used to train a machine learning model, and the classifier uses a Support Vector Machine (SVM). Use grid search with 5-fold cross-validation to find optimal hyperparameters on the training set and retrain on the entire training set. The parameter configurations of the four radiomics models are shown in **Table S8**.

Supplementary Figures


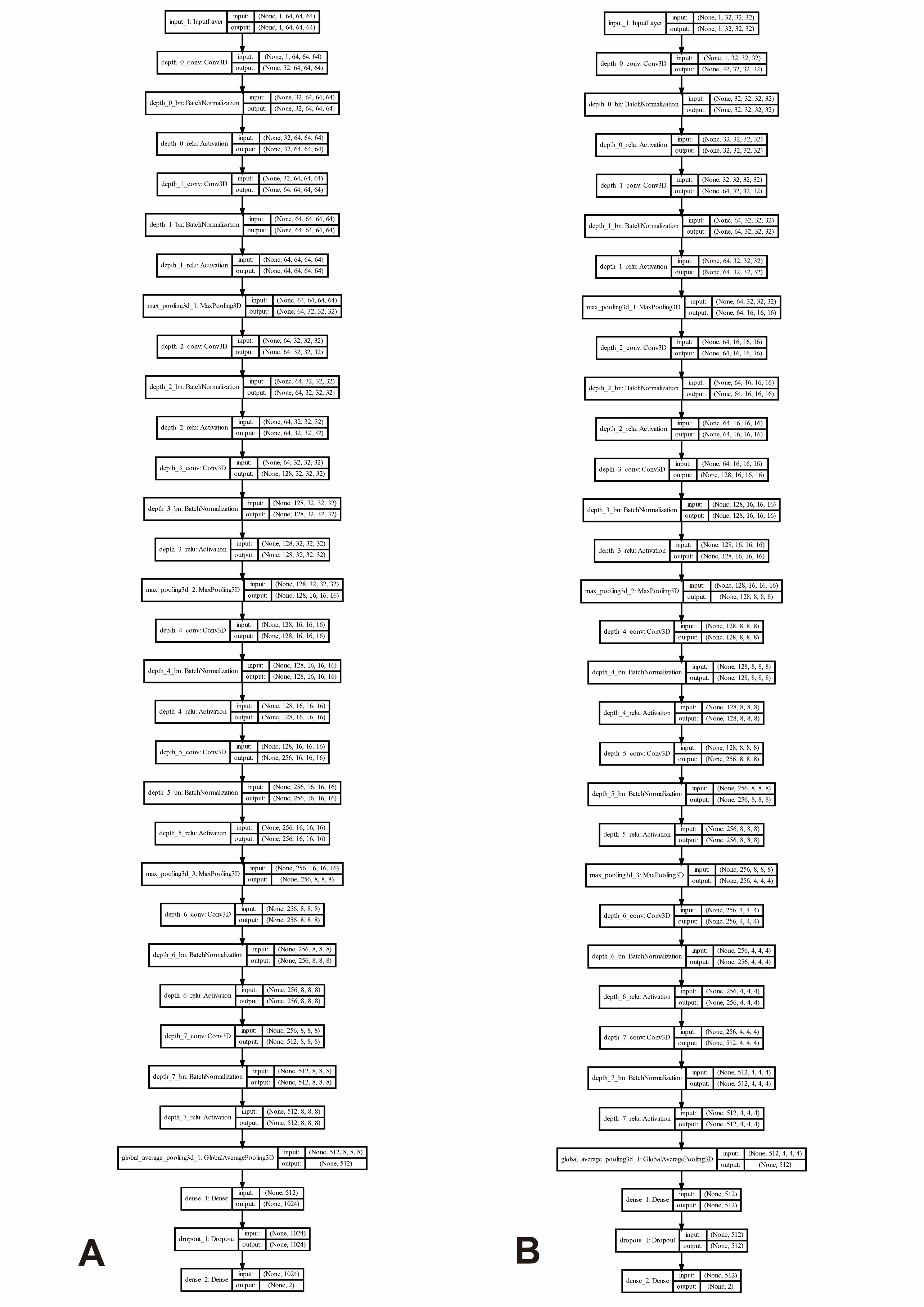


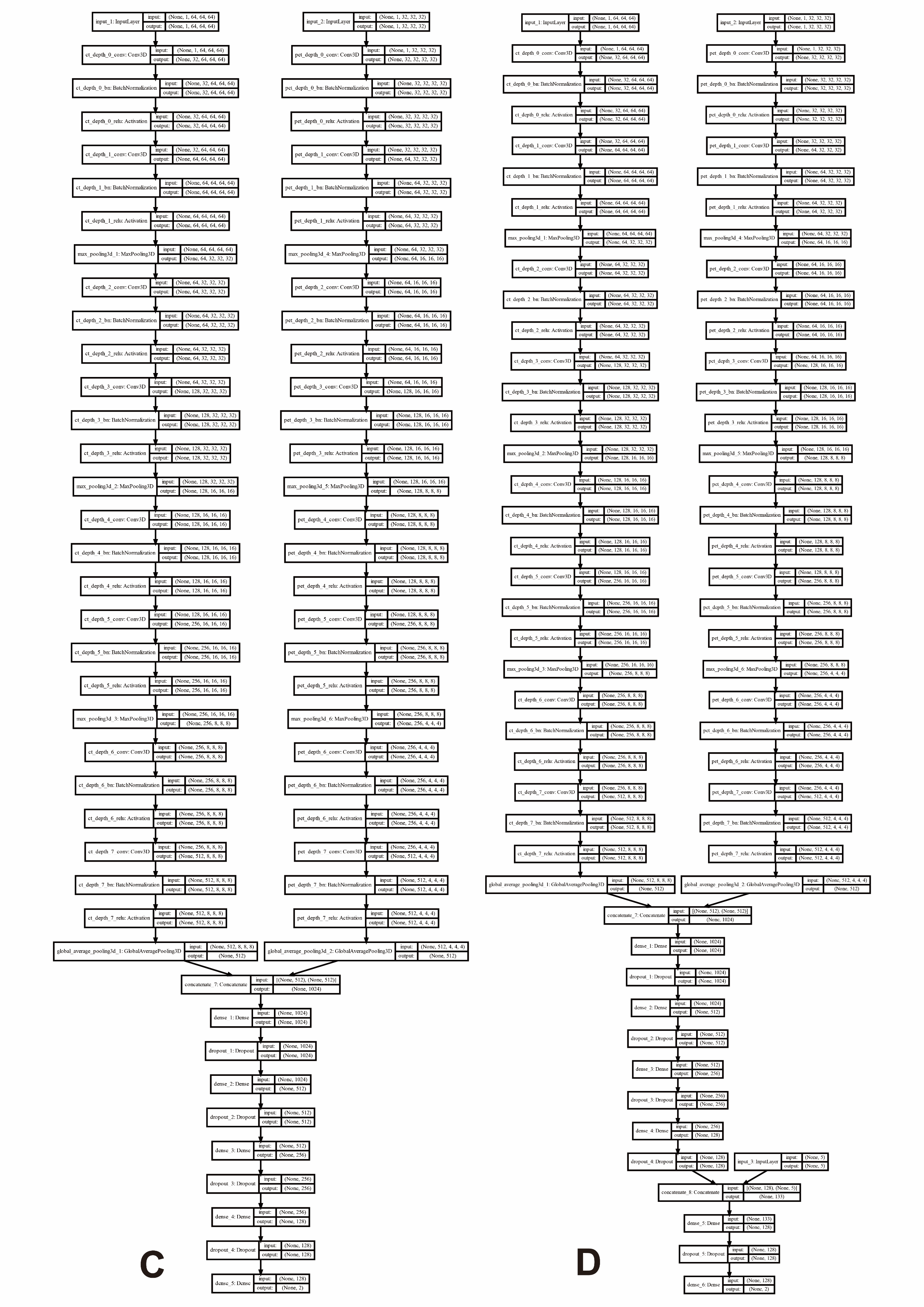


**Figure S1** Schematic diagrams of the four TL models (A: CT_TL; B: PET_TL; C: DS_TL; D: TS_TL). By adding several Dense layers (activation = 'relu') and Dropout layers (rate = 0.5) on the basis of Models Genesis, we aimed to capture the mappings between image features and EGFR mutation labels. Ultimately, a Dense layer of two neurons (activation = 'softmax') was used as the output of the whole model to generate predicted probabilities of the model for different EGFR mutation statuses. Compared to CT_TL and PET_TL, the two models from scratch (CT_origin and PET_origin) do not load pretrained weights and remove Dropout layers (not shown).


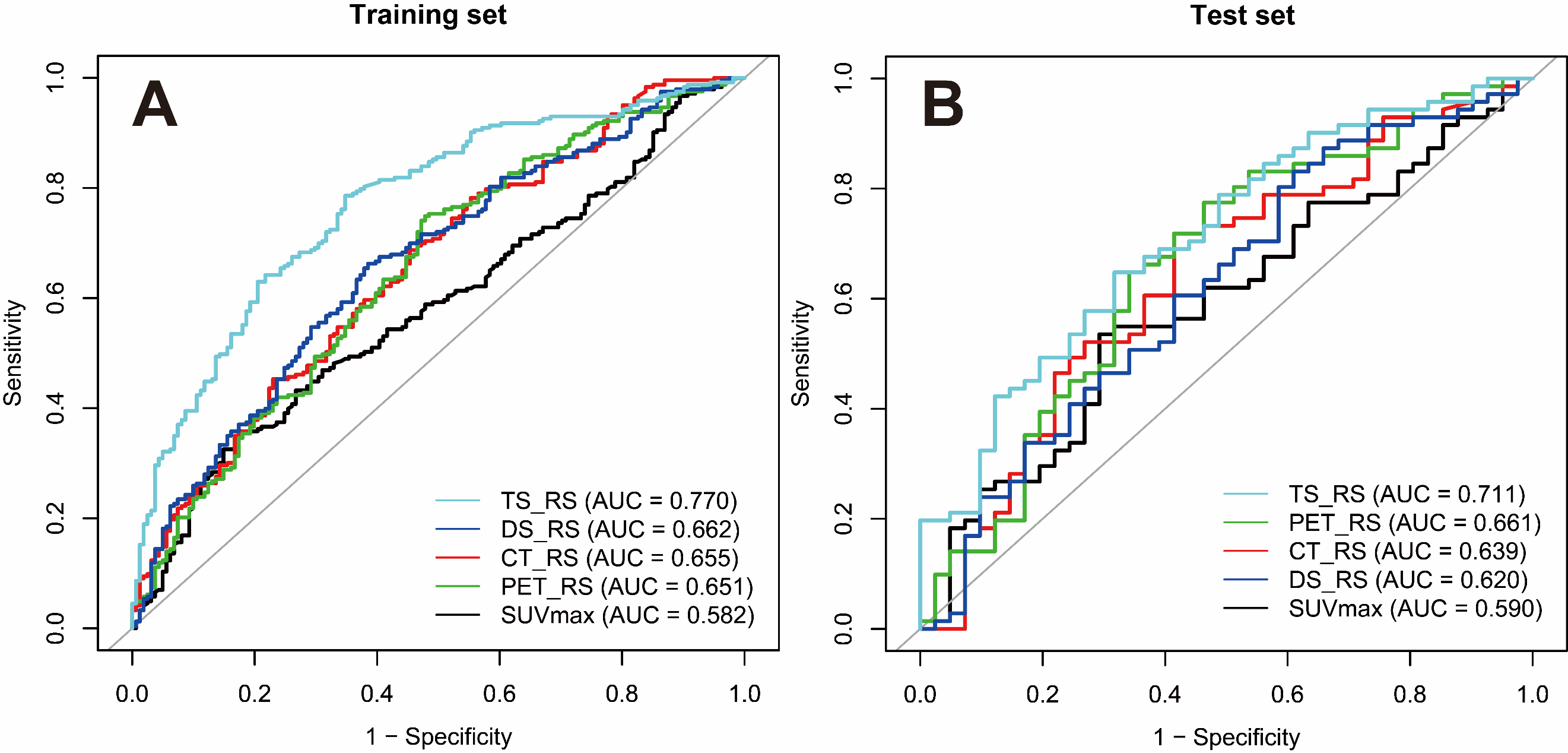


**Figure S2** ROC curves of four radiomics models and SUVmax in training and test sets. AUC, area under the receiver operating characteristic curve; CT_RS, CT radiomics; PET_RS, PET radiomics; DS_RS, PET/CT radiomics; TS_RS, PET/CT radiomics combined with clinical features.

**Supplementary tables**

**Table S1. CheckList for EvaluAtion of Radiomics research (CLEAR checklist)**

| **Section** | **No** | **Item** | **Yes** | **No** | **n/a** | **Page** |
| --- | --- | --- | --- | --- | --- | --- |
| **Title** | | | | | | |
|  | 1 | Relevant title, specifying the radiomic methodology | ☑ | ☐ | ☐ | 1 |
| **Abstract** | | | | | | |
|  | 2 | Structured summary with relevant information | ☑ | ☐ | ☐ | 1-2 |
| **Keywords** | | | | | | |
|  | 3 | Relevant keywords for radiomics | ☑ | ☐ | ☐ | 2 |
| **Introduction** | | | | | | |
|  | 4 | Scientific or clinical background | ☑ | ☐ | ☐ | 4 |
|  | 5 | Rationale for using a radiomic approach | ☑ | ☐ | ☐ | 4-5 |
|  | 6 | Study objective(s) | ☑ | ☐ | ☐ | 5 |
| **Method** | | | | | | |
| *Study Design* | 7 | Adherence to guidelines or checklists (e.g., CLEAR checklist) | ☑ | ☐ | ☐ | 5 |
|  | 8 | Ethical details (e.g., approval, consent, data protection) | ☑ | ☐ | ☐ | 6 |
|  | 9 | Sample size calculation | ☑ | ☐ | ☐ | 6 |
|  | 10 | Study nature (e.g., retrospective, prospective) | ☑ | ☐ | ☐ | 5 |
|  | 11 | Eligibility criteria | ☑ | ☐ | ☐ | 6 |
|  | 12 | Flowchart for technical pipeline | ☑ | ☐ | ☐ | 8 |
| *Data* | 13 | Data source (e.g., private, public) | ☑ | ☐ | ☐ | 5 |
|  | 14 | Data overlap | ☑ | ☐ | ☐ | 6 |
|  | 15 | Data split methodology | ☑ | ☐ | ☐ | 6, 10 |
|  | 16 | Imaging protocol (i.e., image acquisition and processing) | ☑ | ☐ | ☐ | 7 |
|  | 17 | Definition of non‑radiomic predictor variables | ☑ | ☐ | ☐ | 6 |
|  | 18 | Definition of the reference standard (i.e., outcome variable) | ☑ | ☐ | ☐ | 7 |
| *Segmentation* | 19 | Segmentation strategy | ☑ | ☐ | ☐ | 8 |
|  | 20 | Details of operators performing segmentation | ☑ | ☐ | ☐ | 8, S3 |
| *Pre-processing* | 21 | Image pre‑processing details | ☑ | ☐ | ☐ | S1, S3 |
|  | 22 | Resampling method and its parameters | ☑ | ☐ | ☐ | S1, S3 |
|  | 23 | Discretization method and its parameters | ☑ | ☐ | ☐ | S3 |
|  | 24 | Image types (e.g., original, filtered, transformed) | ☑ | ☐ | ☐ | S3 |
| *Feature extraction* | 25 | Feature extraction method | ☑ | ☐ | ☐ | S3-S4 |
|  | 26 | Feature classes | ☑ | ☐ | ☐ | S3 |
|  | 27 | Number of features | ☑ | ☐ | ☐ | S3 |
|  | 28 | Default configuration statement for remaining parameters | ☑ | ☐ | ☐ | S3 |
| *Data preparation* | 29 | Handling of missing data | ☑ | ☐ | ☐ | 9 |
|  | 30 | Details of class imbalance | ☑ | ☐ | ☐ | S2 |
|  | 31 | Details of segmentation reliability analysis | ☑ | ☐ | ☐ | S4 |
|  | 32 | Feature scaling details (e.g., normalization, standardization) | ☑ | ☐ | ☐ | S4 |
|  | 33 | Dimension reduction details | ☑ | ☐ | ☐ | S4 |
| *Modeling* | 34 | Algorithm details | ☑ | ☐ | ☐ | 8 |
|  | 35 | Training and tuning details | ☑ | ☐ | ☐ | S4 |
|  | 36 | Handling of confounders | ☑ | ☐ | ☐ | 10-11 |
|  | 37 | Model selection strategy | ☑ | ☐ | ☐ | S4 |
| *Evaluation* | 38 | Testing technique (e.g., internal, external) | ☑ | ☐ | ☐ | 6, S12-S13 |
|  | 39 | Performance metrics and rationale for choosing | ☑ | ☐ | ☐ | 9 |
|  | 40 | Uncertainty evaluation and measures (e.g., confidence intervals) | ☑ | ☐ | ☐ | 9 |
|  | 41 | Statistical performance comparison (e.g., DeLong’s test) | ☑ | ☐ | ☐ | 9 |
|  | 42 | Comparison with non‑radiomic and combined methods | ☑ | ☐ | ☐ | 8 |
|  | 43 | Interpretability and explainability methods | ☑ | ☐ | ☐ | 8-9 |
| **Results** | | | | | | |
|  | 44 | Baseline demographic and clinical characteristics | ☑ | ☐ | ☐ | 9-10 |
|  | 45 | Flowchart for eligibility criteria | ☑ | ☐ | ☐ | 6 |
|  | 46 | Feature statistics (e.g., reproducibility, feature selection) | ☑ | ☐ | ☐ | S3, S14-S15 |
|  | 47 | Model performance evaluation | ☑ | ☐ | ☐ | 10-11 |
|  | 48 | Comparison with non‑radiomic and combined approaches | ☑ | ☐ | ☐ | 10-11 |
| **Discussion** | | | | | | |
|  | 49 | Overview of important findings | ☑ | ☐ | ☐ | 13-14 |
|  | 50 | Previous works with differences from the current study | ☑ | ☐ | ☐ | 14-16 |
|  | 51 | Practical implications | ☑ | ☐ | ☐ | 17 |
|  | 52 | Strengths and limitations (e.g., bias and generalizability issues) | ☑ | ☐ | ☐ | 16-17 |
| **Open Science** | | | | | | |
| *Data availability* | 53 | Sharing images along with segmentation data [n/e] | ☑ | ☐ | ☐ | 5 |
|  | 54 | Sharing radiomic feature data | ☑ | ☐ | ☐ | 5 |
| *Code availability* | 55 | Sharing pre‑processing scripts or settings | ☑ | ☐ | ☐ | 5 |
|  | 56 | Sharing source code for modeling | ☑ | ☐ | ☐ | 5 |
| *Model availability* | 57 | Sharing final model files | ☑ | ☐ | ☐ | 5 |
|  | 58 | Sharing a ready‑to‑use system [n/e] | ☐ | ☑ | ☐ |  |

**Yes**, details provided; **No**, details not provided; **n/e**, not essential; **n/a**, not applicable

**Table S2. Conventional chest CT and PET/CT image acquisition parameters**

| Acquisition and reconstruction | \| **Acquisition parameters** \| **Siemens Biograph mCT 64** \| \| \| --- \| --- \| --- \| \| **PET** \| **CT** \| \| **^18^F-FDG activity (MBq)*** \| 350-550 (3.70-7.77MBq/kg) \| – \| \| **Min/bed position** \| 2.5 \| – \| \| **Crystal** \| LSO \| – \| \| **Reconstruction** \| OSEM + PSF + TOF \| – \| \| **Matrix (pixels)** \| 200×200 \| 512×512 \| \| **Resolution (mm)** \| 4.07 \| 0.78 \| \| **Slice thickness (mm)** \| 3.00mm \| 3.00mm \| \| **Slices** \| – \| 64 \| \| **Voltage (kV)** \| – \| 140 \| \| **Tube current (mA)** \| – \| 64 \| \| **Reconstruction** \| Gaussian filtering with a full-width at half maximum of 2.0mm; 2 iterations and 21 subsets \| B70f very sharp \| \| *Administered activity was calculated according to the European Association of Nuclear Medicine (EANM) guidelines, version 1.0, and from February 2015, version 2.0 \| \| \| |
| --- | --- | --- | --- | --- | --- | --- | --- | --- | --- | --- | --- | --- | --- | --- | --- | --- | --- | --- | --- | --- | --- | --- | --- | --- | --- | --- | --- | --- | --- | --- | --- | --- | --- | --- | --- | --- | --- | --- | --- | --- | --- | --- |

**Table S3. Predictive performance of several deep learning models in the training set**

| Model | AUC (95%CI) | Accuracy | Sensitivity | Specificity | PPV | NPV |
| --- | --- | --- | --- | --- | --- | --- |
| CT_origin | 0.682 (0.630–0.735) | 0.609 | 0.613 | 0.602 | 0.700 | 0.508 |
| CT_TL | 0.739 (0.690–0.789) | 0.678 | **0.802** | 0.491 | 0.704 | 0.622 |
| PET_origin | 0.619 (0.565–0.674) | 0.582 | 0.642 | 0.491 | 0.655 | 0.476 |
| PET_TL | 0.770 (0.723–0.817) | 0.668 | 0.588 | 0.789 | 0.808 | 0.559 |
| DS_TL | 0.823 (0.781–0.864) | 0.757 | 0.782 | 0.720 | 0.809 | 0.686 |
| TS_TL | **0.883 (0.849–0.917)** | **0.807** | 0.798 | **0.820** | **0.870** | **0.729** |

Note: AUC, area under the receiver operating characteristic curve; PPV, positive predictive value; NPV, negative predictive value; CT_origin, CT model from scratch; CT_TL, CT transfer learning; PET_origin, PET model from scratch; PET_TL, PET transfer learning; DS_TL, dual‑stream transfer learning; TS_TL, three-stream transfer learning. Bold numbers indicate the best results for each evaluation metric.

**Table S4. Comparison of clinical characteristics of patients in training and test sets**

|  | Training Set | Test Set | *p*-value |
| --- | --- | --- | --- |
|  | *n* = 404 | *n* = 112 |  |
| Age (years) | 64.1 (9.2) | 63.5 (9.2) | 0.557 |
| Gender |  |  | 0.238 |
| Female | 213 (52.7%) | 52 (46.4%) |  |
| Male | 191 (47.3%) | 60 (53.6%) |  |
| Smoking history | 141 (34.9%) | 34 (30.4%) | 0.369 |
| Type of nodules |  |  | 0.563 |
| Solid | 275 (68.1%) | 73 (65.2%) |  |
| Subsolid | 129 (31.9%) | 39 (34.8%) |  |
| Location of nodules |  |  | 0.161 |
| Upper right | 128 (31.7%) | 31 (27.7%) |  |
| Middle right | 20 (5.0%) | 12 (10.7%) |  |
| Lower right | 84 (20.8%) | 26 (23.2%) |  |
| Upper left | 107 (26.5%) | 30 (26.8%) |  |
| Lower left | 65 (16.1%) | 13 (11.6%) |  |
| Tumor long axis (mm) | 27.0 (20.4-40.5) | 33.0 (24.6-45.9) | 0.009 |
| Tumor short axis (mm) | 19.7 (14.6-29.8) | 25.3 (16.9-31.0) | 0.016 |
| Clinical stage |  |  | 0.009 |
| I | 177 (43.8%) | 33 (29.5%) |  |
| II | 15 (3.7%) | 9 (8.0%) |  |
| III | 68 (16.8%) | 17 (15.2%) |  |
| IV | 144 (35.6%) | 53 (47.3%) |  |
| CEA (ng/ml) | 4.08 (1.90-13.00) | 5.18 (2.32-13.81) | 0.219 |
| SUVmax | 11.29 (4.53-17.68) | 14.57 (6.43-18.99) | 0.042 |
| EGFR |  |  | 0.534 |
| Wild-type | 161 (39.9%) | 41 (36.6%) |  |
| Mutation | 243 (60.2%) | 71 (63.4%) |  |

Note: Data in the table were expressed as Mean (SD) or Median (Q1-Q3) / N (%).

**Table S5. Predictive performance of the four TL models in different tumor stages**

|  | stage I-II*n* = 234 | stage III-IV*n* = 282 |
| --- | --- | --- |
| Model | AUC (95%CI) | AUC (95%CI) |
| Training set |  |  |
| CT_TL | 0.764 (0.690 - 0.839) | 0.702 (0.631 - 0.773) |
| PET_TL | 0.749 (0.676 - 0.822) | 0.782 (0.721 - 0.843) |
| DS_TL | 0.862 (0.803 - 0.922) | 0.812 (0.754 - 0.870) |
| TS_TL | 0.903 (0.856 - 0.951) | 0.871 (0.823 - 0.919) |
| Test set |  |  |
| CT_TL | 0.661 (0.469 - 0.853) | 0.707 (0.571 - 0.842) |
| PET_TL | 0.650 (0.454 - 0.846) | 0.618 (0.478 - 0.759) |
| DS_TL | 0.708 (0.538 - 0.879) | 0.726 (0.599 - 0.853) |
| TS_TL | 0.667 (0.492 - 0.842) | 0.760 (0.638 - 0.881) |

Note: AUC, area under the receiver operating characteristic curve; CT_TL, CT transfer learning; PET_TL, PET transfer learning; DS_TL, dual‑stream transfer learning; TS_TL, three-stream transfer learning. Bold numbers indicate the best results for each evaluation metric.

**Table S6. Comparison of the prediction performance of deep learning models and radiomics models in the training set**

| Model | AUC (95%CI) | Accuracy | Sensitivity | Specificity | PPV | NPV |
| --- | --- | --- | --- | --- | --- | --- |
| CT_RS | 0.655 (0.601–0.709) | 0.649 | **0.934** | 0.217 | 0.643 | **0.686** |
| CT_TL | 0.739 (0.690–0.789) | 0.678 | 0.802 | 0.491 | 0.704 | 0.622 |
| PET_RS | 0.651 (0.596–0.706) | 0.651 | 0.733 | 0.528 | 0.701 | 0.567 |
| PET_TL | 0.770 (0.723–0.817) | 0.668 | 0.588 | 0.789 | 0.808 | 0.559 |
| DS_RS | 0.662 (0.609–0.716) | 0.636 | 0.827 | 0.348 | 0.657 | 0.571 |
| DS_TL | 0.823 (0.781–0.864) | 0.757 | 0.782 | 0.720 | 0.809 | 0.686 |
| TS_RS | 0.771 (0.724–0.817) | 0.705 | 0.724 | 0.677 | 0.772 | 0.619 |
| TS_TL | **0.883 (0.849–0.917)** | **0.807** | 0.798 | **0.820** | **0.870** | **0.729** |

Note: AUC, area under the receiver operating characteristic curve; PPV, positive predictive value; NPV, negative predictive value; CT_RS, CT radiomics; CT_TL, CT transfer learning; PET_RS, PET radiomics; PET_TL, PET transfer learning; DS_RS, PET/CT radiomics; DS_TL, dual‑stream transfer learning; TS_RS, PET/CT radiomics combined with clinical features; TS_TL, three-stream transfer learning. Bold numbers indicate the best results for each evaluation metric.

**Table S7. The features used in the four radiomics models (CT_RS, PET_RS, DS_RS, and TS_RS)**

| **Model** | **Features** |
| --- | --- |
| **CT_RS** | CT_original_firstorder_Kurtosis  CT_original_firstorder_Median  CT_original_firstorder_Skewness  CT_log-sigma-1-0-mm-3D_firstorder_Energy  CT_log-sigma-4-0-mm-3D_gldm_DependenceVariance  CT_wavelet-LHL_glrlm_LongRunLowGrayLevelEmphasis  CT_wavelet-HLL_firstorder_Energy  CT_wavelet-HHL_firstorder_Kurtosis |
| **PET_RS** | PET_original_shape_Maximum2DDiameterColumn  PET_log-sigma-0-5-mm-3D_gldm_LargeDependenceLowGrayLevelEmphasis  PET_log-sigma-2-5-mm-3D_glszm_ZoneEntropy  PET_log-sigma-5-0-mm-3D_ngtdm_Coarseness |
| **DS_RS** | CT_original_firstorder_Median  CT_wavelet-HHL_firstorder_Kurtosis  PET_original_shape_Maximum2DDiameterColumn  PET_log-sigma-2-5-mm-3D_glszm_ZoneEntropy |
| **TS_RS** | CT_original_firstorder_Median  CT_wavelet-HHL_firstorder_Kurtosis  PET_original_shape_Maximum2DDiameterColumn  PET_log-sigma-2-5-mm-3D_glszm_ZoneEntropy  gender, smoking history, type of nodules, tumor long axis, tumor short axis |

**Table S8. The parameter configurations used in the four radiomics models**

| **Model** | **Parameter configuration** |
| --- | --- |
| **CT_RS** | C=0.035301649268006  class_weight='balanced'  gamma='auto'  kernel='poly'  probability=True |
| **PET_RS** | C=1.7890528172083877  class_weight='balanced'  gamma='auto'  kernel='rbf'  probability=True |
| **DS_RS** | C=0.21447694386320482  class_weight='balanced'  gamma='auto'  kernel='poly'  probability=True |
| **TS_RS** | C=0.611937953844843  class_weight='balanced'  gamma='auto'  kernel='rbf'  probability=True |

**Table S9. Comparison of classical methods and changes in P+ values before and after TKI treatment for the three cases in Figure 6**

|  |  | Case1 | Case2 | Case3 |
| --- | --- | --- | --- | --- |
| Tumor long axis (mm) | Before treatment | 41.1 | 40.9 | 35.5 |
|  | After treatment | 30.2 | 20.7 | 28.5 |
|  | Change rate | **-26.5%** | **-49.4%** | **-19.7%** |
| Tumor short axis (mm) | Before treatment | 29.7 | 26.7 | 30.5 |
|  | After treatment | 24.6 | 15.9 | 27.9 |
|  | Change rate | **-17.2%** | **-40.4%** | **-8.5%** |
| SUVmax | Before treatment | 14.8 | 11.8 | 3.1 |
|  | After treatment | 10.2 | 3.9 | 1.9 |
|  | Change rate | **-31.1%** | **-67.0%** | **-38.7%** |
| P+ value | Before treatment | 0.462 | 0.299 | 0.373 |
|  | After treatment | 0.679 | 0.485 | 0.620 |
|  | Change rate | **47.0%** | **62.2%** | **66.2%** |

**References**

1. Kim HE, Cosa-Linan A, Santhanam N, Jannesari M, Maros ME, Ganslandt T. Transfer learning for medical image classification: a literature review. BMC medical imaging. 2022;22(1):69.

2. Ganeshan B, Panayiotou E, Burnand K, Dizdarevic S, Miles K. Tumour heterogeneity in non-small cell lung carcinoma assessed by CT texture analysis: a potential marker of survival. European radiology. 2012;22(4):796-802.

3. Pedregosa F, Varoquaux G, Gramfort A, Michel V, Thirion B, Grisel O, et al. Scikit-learn: Machine learning in Python. the Journal of machine Learning research. 2011;12:2825-30.
